# Measuring physical, cognitive, and emotional aspects of exhaustion with the BOSS II-short version – results from a representative population-based study in Germany

**DOI:** 10.1186/s12889-022-12961-z

**Published:** 2022-03-24

**Authors:** Antonia M. Werner, Bjarne Schmalbach, Markus Zenger, Elmar Brähler, Andreas Hinz, Johannes Kruse, Hanna Kampling

**Affiliations:** 1 Department of Psychosomatic Medicine and Psychotherapy, University Medical Center of the Johannes Gutenberg University Mainz, Untere Zahlbacher Straße 8, 55131 Mainz, Germany; 2 Department of Personality Psychology and Psychological Assessment, Goethe-University Frankfurt, Frankfurt, Germany; 3 Medical Psychology and Medical Sociology, University Medical Center of the Johannes Gutenberg University Mainz, Mainz, Germany; 4 Faculty of Applied Human Studies, University of Applied Sciences Magdeburg and Stendal, Magdeburg, Germany; 5 Integrated Research and Treatment Center Adiposity Diseases, Behavioral Medicine Unit, Department of Psychosomatic Medicine and Psychotherapy, Leipzig University Medical Center, Leipzig, Germany; 6 Department of Medical Psychology and Medical Sociology, University of Leipzig, Leipzig, Germany; 7 Department of Psychosomatic Medicine and Psychotherapy, Justus Liebig University Giessen, Giessen, Germany; 8 Department of Psychosomatic Medicine and Psychotherapy, Philipps University Marburg, Marburg, Germany

**Keywords:** (psychological) burnout, Exhaustion, Assessment, General population, Public health

## Abstract

**Background:**

The aim of the present study was the construction and psychometric evaluation of a shortened version of the Burnout Screening Scales II (BOSS II), a measure for exhaustion and burnout.

**Methods:**

To this end, among a representative sample of the German general population (*N* = 2429, 52.9% women), we shortened the scale from 30 to 15 items applying ant-colony-optimization, and calculated item statistics of the short version (BOSS II-short). To estimate its reliability, we used McDonald’s Omega (ω). To demonstrate validity, we compared the correlation between the BOSS II-short and the BOSS II, as well as their associations with depression, anxiety, and quality of life. Furthermore, we evaluated model fit and measurement invariance across respondent age and gender in confirmatory factor analyses (CFA). Finally, we present adapted norm values.

**Results:**

The CFA showed an excellent model fit (χ^2^ = 223.037, *df* = 87, *p *< .001; CFI = .975; TLI = .970; RMSEA [90%CI] = .036 [.031;.040]) of the BOSS II-short, and good to very good reliability of the three subscales: ‘physical’ (ω = .76), ‘cognitive’ (ω = .89), and ‘emotional’ (ω = .88) symptoms. There was strict measurement invariance for male and female participants and partial strict invariance across age groups. Each subscale was negatively related to quality of life (‘physical’: *r* = −.62; ‘cognitive’: *r* = −.50; ‘emotional’: *r* = −.50), and positively associated with depression (‘physical’: *r* = .57; ‘cognitive’: *r* = .67; ‘emotional’: *r* = .73) and anxiety (‘physical’: *r* = .50; ‘cognitive’: *r* = .63; ‘emotional’: *r* = .71).

**Conclusions:**

Overall, the BOSS II-short proved to be a valid and reliable instrument in the German general population allowing a brief assessment of different symptoms of exhaustion. Norm values can be used for early detection of exhaustion.

**Supplementary Information:**

The online version contains supplementary material available at 10.1186/s12889-022-12961-z.

## Background

Various constructs and definitions of burnout have been internationally published. Maslach and Leiter [1] summarized burnout to be the reflection of a ‘breakdown in the relationship of people with their work’ [2], and hence, established a clear link between burnout and occupation. In contrast, other sources consider burnout to be a medical condition and assume a conceptual confusion of burnout and depressive disorders, and therefore, raising the question whether burnout can be considered as a distinct construct or rather a specific aspect of depressive disorders in terms of a burnout-depression overlap [3–12]. The conceptual inconsistencies regarding the definition and diagnosis of burnout are summarized by Mäkikangas and Kinnunen [13] as well as the Health Technology Assessment (HTA) Report [14]. The HTA report concluded that the great heterogeneity across studies and theoretical frameworks (e.g, determining the types, development or progression of symptoms) do not allow for a standardized, universal, and internationally accepted diagnosis of burnout. Disregarding (or possibly ending) this controversy only very recently, the World Health Organization (WHO) decided on a burnout definition, and launched an announcement stipulating the inclusion of burnout in the 11th revision of the International Classification of Diseases (ICD-11) as an occupational phenomenon and not a medical condition [15]. In this framework, burnout is characterized by three dimensions: 1) ‘feelings of energy depletion or exhaustion’, 2) ‘increased mental distance from one’s job, or feelings of negativism or cynicism related to one’s job’, and 3) ‘reduced professional efficacy’. Thus, burnout represents a factor influencing the health status that ‘refers specifically to phenomena in the occupational context’ [15]. Even if burnout itself will not be considered an illness or a health condition, it has a negative impact for various occupational professions [1, 4, 12, 16–19]. A recent systematic review significantly associated the presence of burnout with a variety of adverse physical (e.g., coronary heart disease, diabetes, prolonged fatigue, hospitalization, pain), psychological (e.g., depressive symptoms, insomnia), and occupational consequences (e.g., absenteeism, job dissatisfaction, job demands, new disability pension) [20].

Apart from the discussion about the description of burnout as an occupational stress syndrome, the burnout facet of ‘feelings of energy depletion or exhaustion’ is a very unspecific stress symptom and commonly observable in other than occupational contexts, and therefore, not limited to employed or self-employed populations. In fact, exhaustion – and fatigue as well – represents a transdiagnostic phenomenon, observable in several physical (e. g. cancer) or mental health conditions (e. g. major depression or somatoform disorders). For example, fatigue is a common disease- and treatment-related symptom among cancer patients [21, 22], and lower quality of life in association with feelings of fatigue is observable in the general population [23]. Therefore, the accurate assessment of exhaustion is not only of importance in screening for the burnout syndrome but rather several kinds of stress-related health issues.

### Assessment of burnout and exhaustion

The most frequently used psychometric tool to assess exhaustion in the context of burnout is the Maslach Burnout Inventory (MBI) [24] comprising 22 items. The three main scales ‘emotional exhaustion’ (nine items), ‘depersonalization’ (five items), and ‘personal accomplishment’ (eight items) are in accordance with the current definition of burnout by the WHO [15]. ‘Emotional exhaustion’ assesses exhaustion at work (e.g. ‘I feel frustrated by my job.’), ‘depersonalization’ measures to what extent individuals are distancing themselves mentally from the own work and people at work (e.g. ‘I feel I treat some recipients as if they were impersonal objects. ’), and ‘personal accomplishment’ asks about how the participants are performing at their work (e.g. ‘I have accomplished many worthwhile things in this job.’). The MBI focuses not only on specific psychosomatic stress symptoms – the exhaustion component of burnout – but considers also other aspects like depersonalization and professional (in)efficacy which can be of consequences not only for the affected individuals, but in case of e.g. health professionals is also related to decreased patients’ safety [25]. However, the MBI was never intended as a diagnostic tool for clinical practice, while, in contrast, the Burnout Screening Scales (BOSS) by Geuenich and Hagemann [26, 27] were developed specifically with the aim to provide a screening tool for clinical practice and to assess clinically relevant symptoms of occupational stress and burnout in the individual, emphasizing additionally other stress components in different areas of life (not only the occupational situation) and psychosomatic symptoms (physical, cognitive, and emotional complaints). Stress-related mental disorders are hardly only a result from chronic stress at work alone but have multiple sources of distress regarding work and family life.

The BOSS comprises three modules: BOSS I, BOSS II, and BOSS III which can be utilized each on their own. The BOSS I asks about stress and complaints, and the BOSS III about resources, each with the four subscales ‘occupation’, ‘own person’, ‘family’, and ‘friends’, referring to the last 3 weeks. In the present study, we focused on the BOSS II which is measuring psychosomatic symptoms regarding different aspects of exhaustion. Compared to the MBI scale ‘emotional exhaustion’, the BOSS II asks specifically about different types of psychosomatic symptoms, covering ‘physical’ (e.g. sleeping problems), ‘cognitive’ (e.g. lower willingness to make decisions) and ‘emotional’ symptoms (e.g. fears about the future).

Areas of application of the BOSS II are occupational medicine, psychotherapy, psychosocial counselling, and general medical care [26]. In all of these settings, it is important to have psychometrically sound screening tools to complement clinical interviews, and assess mental health issues like stress-related exhaustion. Especially at first visits to doctors and therapists, it is helpful to get a reliable and valid overview of the patient’s situation quickly. There must be a balance between breadth and depth of the assessment. The earlier (increasing) exhaustion is recognized, the better it can be addressed in terms of prevention and treatment. As the BOSS-II assesses exhaustion more detailed than the MBI, it can be used not only to measure burnout risk, but elevated exhaustion in general, which is not a burnout-specific symptom but is present in several clinical conditions.

### Study objectives

The present study aimed at the development and psychometric evaluation of a shortened version of the BOSS II to provide an economic measure for the assessment of stress-related physical, cognitive, and emotional symptoms. Based on the original BOSS II with 30 items, the main goal was a version with only 15 items without compromising the psychometric quality of the measure. Beyond that, we explored which groups of the general population report more frequently physical, cognitive, and emotional complaints, respectively.

We expected to find a shorter version of the BOSS-II with comparable psychometric properties to the original scale in terms of factor structure, internal consistency, and a similar correlational pattern. As the BOSS-II assesses physical, cognitive, and emotional symptoms of exhaustion, which are observable in a variety of physical and mental health issues, especially in depression, we expected to find similar differences in terms of gender and age as for major depression in each subscale, with female participants reporting more exhaustion than male participants, and older participants reporting more exhaustion than younger participants. Furthermore, we expected moderate positive associations between the BOSS-II and the mental health conditions of depression and anxiety as well as a moderate negative association with quality of life.

## Methods

### Study procedure

The present study was designed as a cross-sectional study among the general population in Germany. The data collection took place in July and August 2011. It was conducted in cooperation with the independent demography research service USUMA Berlin (Unabhängiger Service für Umfragen, Methoden und Analysen, Berlin, Germany). The aim was to obtain a representative sample of the German general population. USUMA applied a multistage sampling method based on electoral districts, households, and persons in the household. In a first step, German regional areas were predefined using the reference system for representative studies in Germany provided by the ADM-Sampling-System. In this system, the total area of Germany is divided into 258 regions. Based on these regions, 17 target households per region were selected via random route procedures, leading to 4386 contacted households, and household members were randomly selected using the Kish selection grid. Eligibility criteria were sufficient German language skills and an age of ≥14. The survey comprised two written questionnaires. The first questionnaire contained sociodemographic and household information and was conducted face to face with experienced and trained interviewers in order to control for representativeness of the sample. After that, participants answered the second part of the survey independently. In that time, the interviewer was still present and available for questions. All participants gave their informed consent before participation.

### Participants

Of 4386 contacted households and target persons, there was a total response rate of 59%, leading to a total sample of *N* = 2555 participants. We removed all participants who had missing values on at least one of the BOSS’s items from the analysis as well as participants under the age of 18. This led to a final analysis sample size of *N* = 2429. Table 1 provides sociodemographic characteristics of the final sample. The representativeness of the sample in terms of respondents’ age and gender could be confirmed by comparing the distributions with data provided by the Federal Statistical Office of Germany [28].

**Table 1:** Sample characteristics and one-way analyses of variance (ANOVA) for gender, age, education, marital status, and income

			BOSS II-short_physical			BOSS II-short_cognitive			BOSS II-short_emotional		
Gender	*n*	%	*M*	(*SD*)	ANOVA	*M*	(*SD*)	ANOVA	*M*	(*SD*)	ANOVA
Female	1286	52.9	4.10	(4.20)	*F*(1, 2427) = 10.67, *p* = .001, η^2^_p_ = .004	2.78	(3.99)	F(1, 2427) = 8.09, *p* = .001, η^2^_p_ = .003	3.38	(4.29)	*F*(1, 2427) = 14.78, *p* < .001, η^2^_p_ = .006
Male	1143	47.1	3.55	(3.95)		3.55	(3.95)		3.55	(3.95)	
Age (*M* = 50.35; *SD* = 17.44)											
18-29 years	365	15.0	1.66	(2.89)	*F*(5, 2423) = 88.88, *p* < .001, η^2^_p_ = .155	1.68	(2.95)	*F*(5, 2423) = 24.69, *p* < .001, η^2^_p_ = .048	2.42	(3.57)	*F*(5, 2423) = 6.43, *p* < .001, η^2^_p_ = .013
30-39 years	334	13.8	2.13	(3.01)		1.89	(3.33)		2.67	(3.67)	
40-49 years	481	19.8	3.22	(3.61)		2.38	(3.68)		3.04	(4.16)	
50-59 years	447	18.4	4.10	(4.15)		2.48	(3.83)		3.42	(4.41)	
60-69 years	393	16.2	5.13	(4.05)		2.54	(3.55)		2.90	(3.92)	
≥ 70 years	409	16.8	6.41	(4.41)		4.30	(4.46)		3.87	(4.52)	
Education											
≤ 9 years	1038	42.7	4.76	(4.24)	*F*(3, 2425) = 33.91, *p* < .001, η^2^_p_ = .040	3.16	(4.21)	*F*(3, 2425) = 15.41, *p* < .001, η^2^_p_ = .019	3.51	(4.35)	*F*(3, 2425) = 7.32, *p* < .001, η^2^_p_ = .009
10 years	880	36.2	3.26	(3.93)		2.11	(3.40)		2.85	(4.00)	
≥ 11 years	503	20.7	2.98	(3.67)		2.23	(3.31)		2.63	(3.74)	
School students	8	0.3	0.63	(1.06)		0.25	(0.46)		1.25	(1.49)	
Marital status											
Married	1210	49.8	3.77	(3.90)	*F*(5, 2423) = 37.32, *p* < .001, η^2^_p_ = .072	2.20	(3.37)	*F*(5, 2423) = 20.29, *p* < .001, η^2^_p_ = .040	2.53	(3.59)	*F*(5, 2423) = 14.92, *p* < .001, η^2^_p_ = .030
Committed relationship	129	5.3	2.25	(3.19)		2.08	(3.50)		2.97	(4.52)	
Single	454	18.7	2.57	(3.77)		2.22	(3.55)		3.01	(4.07)	
Separated	39	1.6	3.54	(4.16)		3.13	(4.17)		4.18	(4.66)	
Divorced	300	12.4	4.34	(4.16)		2.85	(4.16)		4.02	(4.58)	
Widowed	297	12.2	6.28	(4.45)		4.50	(4.66)		4.42	(4.87)	
Employment											
Working full-time	981	40.4	2.53	(3.19)	*F*(4, 2421) = 111.62, *p* < .001, η^2^_p_ = .156	1.80	(3.13)	*F*(4, 2421) = 33.18, *p* < .001, η^2^_p_ = .052	2.35	(3.46)	*F*(4, 2421) = 20.65, *p* < .001, η^2^_p_ = .033
Working part-time	264	10.9	3.17	(3.40)		2.22	(3.33)		3.19	(4.04)	
Unemployed	293	12.1	4.11	(4.24)		3.11	(4.44)		4.32	(4.85)	
Retired	753	31.0	6.09	(4.48)		3.70	(4.25)		3.68	(4.52)	
In training	135	5.6	1.55	(2.69)		1.53	(2.75)		2.17	(3.28)	
Missing	3	0.1									
Monthly net income											
< 1500€	892	36.7	4.79	(4.59)	*F*(3, 2425) = 33.52, *p* < .001, η^2^_p_ = .040	3.35	(4.34)	*F*(3, 2425) = 24.60, *p* < .001, η^2^_p_ = .030	4.06	(4.71)	*F*(3, 2425) = 31.75, *p* < .001, η^2^_p_ = .038
< 2500€	784	32.3	3.73	(3.84)		2.43	(3.53)		2.86	(3.95)	
≥ 2500€	683	28.1	2.80	(3.42)		1.75	(2.96)		2.13	(3.22)	
Refused to answer	70	2.9	2.96	(3.39)		2.26	(4.04)		2.36	(3.22)	

### Instruments

The Burnout Screening Scales II (BOSS II) [26, 27] consist of 30 items addressing burnout associated physical (‘I suffer from sleep disorders.’), cognitive (‘My willingness to make decisions has been lost.’), and emotional symptoms (‘I have fear of the future.’). Items are evenly distributed across domains (ten items each). The BOSS II asks respondents to what extent they suffered from any of the symptoms during the last 7 days ranging from 0 (‘does not apply’) to 5 (‘applies fully’). In the original 30-item version, internal consistency, calculated for different samples, ranged between α = .79 and α = .88 for ‘physical’, between α = .78 and α = .97 for ‘cognitive’, and between α = .81 and α = .96 for ‘emotional’ symptoms [26]. For each of these subscales, it is possible to build three types of values: total score, intensity value, and width value. In the current study, we did all calculations with the total score of each scale.

The European Quality of Life Scale (EuroQol) in its revised version 5 L was used to assess health-related quality of life [29]. It consists of five items – utilizing five-point Likert scales with various wordings – measuring the extent to which respondents experience limitations in their daily life based on health issues. By reverse-coding, a quality of life index is obtained. Based on the sample of the present study, the coefficient of ω = .88 indicated good reliability.

To assess symptoms of depression, we used the PHQ-9 [30–32] depression module of the Patient Health Questionnaire (PHQ) [33]. It consists of nine items scoring from 0 (‘not at all’) to 3 (‘nearly every day’). In the present sample, internal consistency was high (ω = .91).

The Generalized Anxiety Disorder Scale-7 (GAD-7) [34, 35] is a brief measure for assessing generalized anxiety disorder and severity of general anxiety symptoms. It contains seven items ranging from 0 (‘not at all’) to 3 (‘nearly every day’). In the present sample, reliability was high (ω = .90).

Relevant sociodemographic parameters were gender (male or female), age, education (≤ 9 years, 10 years, ≥ 11 years), marital status (married, committed relationship, single, separated, divorced, widowed), employment (working full-time, working part-time, unemployed, retired, in training), and monthly net income (≤ 1500 €, < 2500 €, ≥ 2500 €), assessed in accordance with the demographic standards of the Federal Statistical Office of Germany.

### Data analysis

All analyses were conducted using R [36]. Applied packages were lavaan [37], psych [38], semTools [39], and stuart [40]. First, we randomly split our full sample into an exploratory (*n* = 1197) and a confirmatory one (*n* = 1232). In order to reduce the initial item pool of 30 items while retaining the three-factor structure, we used the R package stuart [40] among the exploratory subsample. Stuart uses ant-colony-optimization to construct subsets of possible items and compares them to find the optimal model solution – in terms of model fit and reliability – for a given number of items and factors. We constrained the search algorithm to look for three-factorial solutions with five items per factor, and to prefer solutions that are invariant across respondent gender.

The solution generated by stuart was tested in the confirmatory subsample, using confirmatory factor analysis (CFA) with robust maximum likelihood estimation (MLM) and robust formulas for estimating fit indices [41, 42]. To evaluate model fit, we referred to the χ^2^-test, interpreting χ^2^ as stated by Hu and Bentler [43] as well as Schermelleh-Engel et al. [44], according whom, χ^2^ should ideally be non-significant, and the ratio of χ^2^and degrees of freedom (*df*) should be smaller than 2 (or 3) to indicate good (or acceptable fit). However, as these statistics are biased by sample size, we relied additionally on the following indices in evaluating model fit: the Comparative Fit Index (CFI) and the Tucker-Lewis Index (TLI) which should both be greater than .95, the Root Mean Square Error of Approximation (RMSEA) and its 90% confidence interval which shouldbe smaller than .05 (or .08) to indicate good (or acceptable) fit, as well as the Standardized Root Mean Square Residual (SRMR) which should be smaller than .05 (or .10) to signify good (or acceptable) fit. We report McDonald’s ω as a measure of internal consistency [45].

To investigate group differences, we conducted one-way Analyses of Variance (ANOVA) in the sociodemographic variables gender, age group in years, education, marital status, employment, and monthly net income. To interpret effect sizes of the results, we used partial eta-squared (η_p_^2^). η_p_^2^ values of .01 can be interpreted as small, values of .06 as medium, and values of .14 as large [46].

Measurement invariance was tested using the customary procedure of comparing increasingly restrictive structural equation models representing increasingly strict levels of invariance: equal factor loadings for metric invariance, equal item intercepts for scalar invariance, and equal residual variances for strict invariance [47]. Differences between models of ≤ .01 in CFI and gamma hat (GH) are evidence for invariance [48]. Only when metric and scalar invariance are met, latent and observed mean scores can be compared reasonably [49]. When strict invariance holds, this further means that all differences in observed variances are caused by differences in latent variances – that is the construct under study [49, 50].

Finally, we report normative percentile values stratified by gender and age group.

## Results

### Development and psychometric properties of the BOSS II-short

As outlined above, we used stuart [40] to find the optimal solution for a short form of the BOSS II. Among all 16,003,008 possible models, the algorithm selected the configuration presented in Table 2. This model had good overall fit: χ^2^(210) = 613.625, χ^2^/*df* = 2.922; *p* < .001, CFI = .961, TLI = .960, RMSEA = .059 (.053; .064), SRMR = .047.

**Table 2:** Item descriptive statistics of the short version of the Burnout Screening Scales II (BOSS II-short)

	*M*	*SD*	γ1	γ2	*r*_it_	λ	ω
BOSS II-short_physical	3.837	4.092	1.424	2.184			.755
Item 5	.459	.966	2.480	6.193	.550	.635	
Item 6	1.200	1.333	.990	.264	.542	.602	
Item 8	.467	.872	2.193	5.142	.516	.630	
Item 9	.862	1.149	1.373	1.436	.586	.682	
Item 10	.849	1.272	1.500	1.391	.567	.585	
BOSS II-short_cognitive	2.576	3.777	1.944	4.168			.888
Item 13	.505	.957	2.245	5.181	.745	.790	
Item 15	.403	.853	2.542	6.902	.750	.809	
Item 16	.566	.912	1.836	3.594	.746	.811	
Item 18	.637	.987	1.619	2.223	.621	.692	
Item 19	.464	.848	2.084	4.488	.767	.828	
BOSS II-short_emotional	3.083	4.110	1.880	3.591			.879
Item 21	.984	1.256	1.278	.973	.637	.662	
Item 23	.448	.915	2.324	5.255	.751	.824	
Item 24	.487	.899	2.102	4.453	.829	.879	
Item 25	.526	.929	1.976	3.752	.791	.870	
Item 26	.639	.919	1.537	2.162	.684	.701	

Note. γ1 = skewness; γ2 = excessive kurtosis; *r*_it_ = corrected item-total correlation; λ = standardized factor loading from the confirmatory factor analysis; ω = reliability coefficient omega

As reported in Table 2, descriptive statistics for all items – in addition to the subscale scores – were good for the most part. The corrected item-total correlations exceeded .500 for all scales, and are thus satisfactory [51]. For 10 of the 15 items, skewness and kurtosis were within the limits of absolute skewness < 2 and absolute excessive kurtosis < 4, provided by Kim [52]. Remaining five items had deviations indicating non-normal distributions. Specifically, we found right-skewness and slight deviations from normal distribution.

To evaluate the BOSS II-short’s factor structure, we tested the fit of the model, which we constructed in the exploratory subsample, also in the confirmatory subsample. Model fit of the 15 items solution can be considered as very good. Detailed statistics of these analyses can be found in Table 3. Standardized factor loadings exceeded .500, and for all but one indicator .600. Factor inter-correlations were high, *r*_physical, cognitive_ = .751, *r*_physical, emotional_ = .687, and *r*_cognitive, emotional_ = .853. These values are somewhat higher than desirable. However, they are still lower than those of the original 30-item BOSS II (*r*_physical, cognitive_ = .767, *r*_physical, emotional_ = .716, *r*_cognitive, emotional_ = .872). Internal consistency was very good, albeit slightly reduced compared to the original BOSS II, ω_physical_ = .858, ω_cognitive_ = .935, and ω_emotional_ = .923. Overall, the model showed very good fit in all measures.

**Table 3:** Model fit indices for the Burnout Screening Scales II (BOSS II), original and short version

	χ^2^	*df*	*p*	χ^2^/*df*	CFI	TLI	RMSEA (90% CI)	SRMR
Three-factor model of the BOSS II, original	1542.938	402	< .001	3.838	.909	.902	.048 (.046; .050)	.044
Three-factor model of the BOSS II-short	223.037	87	< .001	2.564	.975	.970	.036 (.031; .040)	.033

Note. *CFI* Comparative Fit Index, *TLI* Tucker-Lewis-Index, *RMSEA* Root Mean Square Error of Approximation, *SRMR* Standardized Root Mean Square Residual

The results of the ANOVAs comparing different categories of gender, age group, education, marital status, employment status, and monthly net income for the three BOSS II-short subscales are depicted in Table 1. We found statistically significant results for all comparisons. However, regarding the effect sizes, the majority of comparisons show small or negligible effect sizes. There were very small gender differences for all three BOSS II-short subscales with female participants reporting more physical symptoms, and male participants more cognitive and emotional symptoms. Older participants reported more physical and cognitive symptoms. There was a large effect in the subscale physical complaints (η_p_^2^ = .155) for age group, explaining 16% of variance, and a small effect in cognitive symptoms (η_p_^2^ = .048). Regarding employment, for physical symptoms there was a proportion of explained variance of about 16% (η_p_^2^ = .156), with retired and unemployed participants reporting the most symptoms. Looking more closely at different employment groups, retired participants had the highest scores in physical and cognitive symptoms, and unemployed participants reported the most emotional exhaustion. In all three BOSS II-Short subscales, part-time working participants had more symptoms of exhaustion than full-time working persons.

### Measurement invariance of the BOSS II-short

Regarding measurement invariance of the BOSS II-short, the analyses revealed clear evidence for strict invariance across respondent gender with all CFI and GH comparisons revealing very small deviations (see Table 4). In contrast, there were large deviations when considering participant age: we found clear evidence for metric invariance but scalar invariance was only achieved by freeing the intercepts of Items 6 and 10 to vary between groups.

**Table 4:** Tests of measurement invariance of the short version of the Burnout Screening Scales-II (BOSS II-short)

Model	χ^2^ (*df*)	Δχ^2^	Δ*df*	*p*	CFI	ΔCFI	GH	ΔGH
Gender (*n* = 1232)								
Configural invariance	336.628 (174)				.971		.983	
Female	171.851 (87)				.973		.983	
Male	164.823 (87)				.961		.982	
Metric invariance	355.186 (186)	18.558	12	.010	.969	−.002	.982	−.001
Scalar invariance	396.448 (198)	41.262	12	<.001	.965	−.004	.979	−.003
Strict invariance	427.413 (213)	30.964	15	.009	.961	−.004	.977	−.002
Age (*n* = 2429)								
Configural invariance	815.163 (522)				.970		.984	
18-29	141.475 (87)				.957		.980	
30-39	133.281 (87)				.970		.982	
40-49	132.751 (87)				.977		.988	
50-59	122.451 (87)				.979		.989	
60-69	150.234 (87)				.971		.980	
≥ 70	104.335 (87)				.991		.994	
Metric invariance	898.958 (582)	83.795	60	.023	.967	−.003	.983	−.001
Scalar invariance	1208.601 (642)	309.644	60	<.001	.944	−.023	.970	−.013
Partial scalar invariance^a^	1049.089 (632)	150.131	50	<.001	.958	−.009	.978	−.005
Strict invariance^a^	1677.223 (707)	628.134	75	<.001	.904	−.054	.949	−.029
Partial strict invariance^a, b^	1176.248 (672)	127.159	45	<.001	.949	−.009	.973	−.007

Note. ^a^the intercepts of Items 6 and 10 were freed to vary between groups

^b^the residuals of Items 5, 6, 10, 13, 16, 18, and 19 were freed to vary between groups

### Further validity aspects of the BOSS II-short and normative values

Regarding the BOSS II-short’s convergent validity, we found the expected pattern of correlations reported in Table 5. The BOSS II-short – as a measure of exhaustion – correlated positively with symptoms of depression and anxiety, and negatively with quality of life. Moving from the 30-item to the 15-item versions of the BOSS II, we naturally observed a reduction in variance explained. This decline was significant for four of the nine pairs of associations, |Δ*r*| ≤ .042, Δ*z* ≤ 2.57. Yet, the effect sizes were very small, making up less than 1% of overall variance.

**Table 5:** Correlations of the study variables

	1	2	3	4	5	6	7	8	9
1 – BOSS II-short_physical	–	.606	.568	.946	.637	.588	−.621	.567	.497
2 – BOSS II-short_cognitive		–	.763	.654	.971	.786	−.499	.666	.628
3 – BOSS II-short_emotional			–	.618	.790	.965	−.504	.727	.707
4 – BOSS II_physical				–	.684	.637	−.622	.604	.539
5 – BOSS II_cognitive					–	.813	−.523	.705	.665
6 – BOSS II_emotional						–	−.502	.732	.718
7 – Euro QoL							–	−.537	−.477
8 – PHQ-9								–	.816
9 – GAD-7									–

Note. All correlations were highly significant (*p* < .001)

*BOSS II-short* Burnout Screening Scales II 15-item version, *BOSS II* Burnout Screening Scales II, original 30-item version, *Euro QoL* Quality of Life, *PHQ-9* Patient Health Questionnaire-9, *GAD-7* Generalized Anxiety Disorder Scale-7

Finally, we calculated norm values for the BOSS II-Short based on our representative sample. We report percentile ranks categorized by respondent gender and age in the Supplementary Tables 1 and 2 (Additional file 1).

## Discussion

The aim of the present study was the construction and psychometric evaluation of a shortened version of the Burnout Screening Scales II (BOSS II) [26, 27], a measure of physical, cognitive, and emotional symptoms, often occurring with burnout, but also in the context of health issues outside of the occupational context, making exhaustion a transdiagnostic phenomenon. For epidemiological as well as etiological research in health and clinical psychology, psychotherapy as well as psychosomatic medicine, it is vital to understand how different psychosocial phenomena are intertwined, and therefore, they need to be assessed simultaneously. However, vast numbers of instruments and items can be extremely time consuming, burdening or even exhausting for participants in such studies. Hence, researchers are keen to keep the number of items as minimal as possible while still having the highest information output. Therefore, short screening instruments are important when aiming to cover several different topics and questions simultaneously.

The final short version of the BOSS II with 15 items (BOSS II-short) showed excellent model fit for the hypothesized three-factor solution. Each subscale is comprised of five items and show good (‘physical symptoms’) or very good internal consistency (‘cognitive symptoms’, ‘emotional symptoms’). To our knowledge this study is the first to investigate the BOSS II (or a short-form of it) for measurement invariance. Specifically, we found that the measurement model is equivalent for men and women but not across the age spectrum. Invariance in the measurement of the burnout facet ‘exhaustion’ across gender was demonstrated by previous research for various burnout scales [53–56]. Therefore, it is likely that exhaustion is characterized by similar physical, cognitive, and emotional symptoms for male and female participants. In contrast, age-related invariance seems unclear for common burnout [57–60], which is also reflected in the present study: metric invariance held, but scalar invariance was only achieved after relaxing two intercept constraints. However, this is to be expected given the nature of the item content. Both of these items belong to the BOSS II-short ‘physical’ subscale and describe phenomena that have been shown to be generally more common in older populations like joint pains and high blood pressure [61, 62]. Strict invariance was only attainable by releasing constraints for seven residual variances. Thus, the interpretation of the BOSS II-short as strictly invariant across age would be highly questionable. We do, however, find strong evidence for partial scalar invariance which is a sufficient condition for meaningful group mean comparisons. This observation fits with the higher burden of disease in older persons [63].

There was almost no loss in validity when comparing BOSS II-short to the original BOSS II with explained variances greater than .90. Similarly, there were minimal reductions in the associations of the BOSS II-short and external measures (depression, anxiety, and quality of life). Four of the nine differences were significant. However, the very small effect sizes of the differences (*R*^*2*^ < .01) indicate that the long- and short-form of the BOSS II are related to depression, anxiety, and quality of life in similar ways. Therefore, with the BOSS II-short, one can obtain (very close to) the same information by asking only half of the questions.

We observed some differences in symptoms of exhaustion between the categories of the variables gender, age group in years, education, marital status, employment, and monthly net income. These effects were the strongest for the subscale ‘physical’, particularly for age group and employment. As we considered also retired participants who scored the highest for physical and cognitive symptoms, these values are most likely confounded with the age group or the burden of disease of these participants. In Germany, the regular age for retirement is between 63 and 65 years and those retiring earlier do so because of medical or other demanding conditions (e. g. care for family members), prohibiting them to continue working. Beyond that, differences in gender and age could be due to different adaption to the concrete work setting. For example, a longitudinal study investigating activity-based flexible offices (A-FO), that are open-space work settings with a flexible work time and work space organization, indicated, that after changing into a A-FO, employees showed worsened work engagement and increased levels of fatigue [64]. These effects differed between men and women as well as employees of different age. Therefore, subjective evaluation of the work setting and its conditions should help to further understand gender and age differences.

Most interesting is the fact that the unemployed participants reported the highest emotional exhaustion, emphasizing that the BOSS II-short is not limited to the occupational context but to more aspects in life where e.g. the absence of an occupation is a major stress event. This is in line with authors who claimed that workers’ occupational health should not be seen isolated but in context with other factors of stress or individual conditions [65]. Such a perspective could also explain that in our study, part-time working participants were physically, cognitively, and emotionally more exhausted than full-time working persons. This could be due to the fact that beyond occupational duties there are other major life stressors to coordinate such as family life, caregiving, one’s own medical care, or other issues. Beyond that, and throughout our analyses, participants with a lower level of education as well as people with a lower income feel physically, cognitively, and emotionally more exhausted. These observations are in line with research in social epidemiology, where we can find higher burden of disease in older, unemployed, poorer, and less educated people who would therefore need more public support in prevention of mental disorders or physical health conditions [66, 67].

### Strengths and limitations

The major strength of our study is the thorough statistical approach allowing us to successfully shorten the BOSS II from 30 to 15 items without substantial loss of information. We based the calculations on a large sample of the German general population, making it possible to screen the level of exhaustion in the population through gender, age, employment status, marital status, income level, and educational level. While we took a more general look at physical, cognitive, and emotional symptoms of exhaustion throughout the German general population, we did not concretely assess the three criteria of burnout, and hence, cannot make conclusions about the source of the personal exhaustion. With regard to physical symptoms, age-related problems might be a relevant confounder so that results have to be interpreted carefully when looking at the different employment groups. Interpretation of results is further limited by the fact that we investigated the BOSS II-short and the original BOSS in the same sample. There is no reason to expect systematic biases in our analyses but future research should nonetheless aim to confirm the present findings by basing their findings on a sample only applying the new 15 items version of the BOSS II-short. Additionally, it is important to note that the BOSS II-short is only a self-report tool and therefore, it is rather assessing burnout risk respectively risk of clinically relevant exhaustion than the presence of burnout or a mental health condition. An elevated score in the BOSS II-short should be followed by a clinical interview conducted by healthcare personnel in order to be able to establish a diagnosis. Future studies could address this aspect, looking for consistency between self-report (BOSS II-short) and clinical interviews. Furthermore, physical exhaustion could also be assessed with objective medical tests such as slowed reflexes or short-term memory problems. Finally, to address the overlap of burnout risk and exhaustion, a direct comparison with the latest version of the MBI could help could help to understand how the BOSS II-short and the MBI capture different constructs and are applicable to different contexts.

## Conclusion

The BOSS II-short comprising only 15 items has good psychometric properties and can add important insight for both epidemiological research as well as for clinical practice. It is particularly useful because of its brevity – with no information loss compared to the original 30-item version. Additionally, our analyses provided first normative values for physical, cognitive, and emotional symptoms assessed with the BOSS II-short, making it easily accessible for its application in practice.

In summary, the BOSS II-short represents a very efficient and informative assessment tool, economically applicable in large scale surveys or for initial individual assessments in clinical care. Its use in epidemiological research might help to provide a better understanding of public (mental) health.

## Supplementary Information

**Additional file 1: Supplementary Table 1.** Normative percentile ranks for the BOSS II-short for female participants. **Supplementary Table 2.** Normative percentile ranks for the BOSS II-short for male participants.

**Additional file 2:** R Code.

## Data Availability

The data that support the findings of this study are available on reasonable request from the corresponding author [AMW]. The data are not publicly available due to the participants not having given their consent for publishing them in data repositories. We provide the R code as supplementary material (Additional file 2).
