# Supplementary material for: Measuring physical, cognitive, and emotional aspects of exhaustion with the BOSS II-short version – results from a representative population-based study in Germany
Source: BMC Public Health. 2022 Mar 24;22:579. doi: 10.1186/s12889-022-12961-z (PMC8943994; doi:10.1186/s12889-022-12961-z)
Supplement: Supplementary file 1 — Additional file 1: Supplementary Table 1. Normative percentile ranks for the BOSS II-short for female participants. Supplementary Table 2. Normative percentile ranks for the BOSS II-short for male participants. [file 12889_2022_12961_MOESM1_ESM.pdf]

Additional file 1

Supplementary Table 1

*Normative percentile ranks for the BOSS II-short for **female** participants*

	Age in years																	
	18-29 (*n* = 191)			30-39 (*n* = 168)			40-49 (*n* = 255)			50-59 (*n* = 242)			60-69 (*n* = 211)			≥ 70 (*n* = 219)		
Sum Score	P	C	E	P	C	E	P	C	E	P	C	E	P	C	E	P	C	E
0	54	57	39	40	57	34	24	46	32	18	47	28	13	41	35	6	26	21
1	66	68	52	53	66	49	40	59	49	26	56	40	19	55	45	8	36	36
2	79	75	66	70	73	64	52	66	61	41	64	56	26	65	58	16	44	50
3	82	83	72	77	80	73	64	73	68	52	71	65	36	71	68	24	52	58
4	85	88	79	83	85	80	71	78	74	62	74	70	48	76	75	32	57	64
5	88	90	83	88	88	84	78	83	78	69	79	74	57	80	82	39	65	70
6	92	91	87	90	90	86	84	87	81	77	84	79	66	82	84	53	71	74
7	93	93	90	92	91	89	89	90	86	83	86	83	74	88	85	63	75	78
8	95	95	92	93	93	90	91	94	89	86	90	85	80	91	88	70	79	82
9	96	98	93	96	95	91	94	96	91	90	92	89	88	94	91	74	85	85
10	97	98	95	97	96	92	96	97	93	91	93	91	91	95	94	80	87	88
11	97	98	96	97	97	94	97	97	94	93	94	93	93	96	96	86	89	89
12	99	99	96	98	97	96	97	98	95	93	97	94	94	97	97	90	92	91
13	99	99	98	99	98	96	97	98	96	96	98	94	95	97	98	93	93	93
14	99	99	98	100	98	98	98	98	97	97	98	95	95	99	99	93	94	95
15	99	100	99		98	98	98	100	98	98	99	96	96	100	99	95	95	96
16	99		99		99	98	99	100	99	99	99	98	97	100	99	97	96	97
17	99		99		99	98	99		99	100	99	98	98	100	100	97	98	99
18	99		100		99	99	99		100	100	99	99	99	100	100	97	98	99
19	100				99	99	100		100	100	99	99	100	100	100	98	99	99
20					99	99	100			100	100	99	100	100	100	99	99	99
21					99	99	100			100	100	100			100	100	99	99
22					100	99	100			100	100	100			100		99	100
23						100					100	100					99	100
24												100					100	100
25																	100	

Note. P= Physical, C= Cognitive, E= Emotional

Supplementary Table 2

*Normative percentile ranks for the BOSS II-short for **male** participants*

	Age in years																	
	18-29 (*n* = 174)			30-39 (*n* = 166)			40-49 (*n* = 226)			50-59 (*n* = 205)			60-69 (*n* = 182)			≥ 70 (*n* = 190)		
Sum Score	P	C	E	P	C	E	P	C	E	P	C	E	P	C	E	P	C	E
0	60	62	47	43	56	37	30	54	39	22	54	40	12	45	38	11	24	32
1	72	74	63	58	66	51	39	64	51	33	62	54	16	58	54	16	32	45
2	79	80	77	73	75	67	54	72	69	51	70	60	31	69	66	24	44	58
3	84	82	79	81	80	75	64	76	74	60	79	70	42	75	77	33	52	66
4	89	84	84	86	85	81	75	81	79	70	85	76	52	82	82	42	66	76
5	90	86	87	92	93	87	84	85	82	77	88	80	63	87	85	55	73	80
6	91	89	90	94	95	89	86	87	86	82	91	83	73	90	89	63	81	82
7	95	93	91	95	96	93	90	88	88	87	91	86	79	92	90	69	85	86
8	97	93	91	96	96	94	92	91	89	90	93	89	87	93	91	76	91	88
9	98	95	93	96	96	96	94	93	91	91	93	91	90	95	93	81	93	90
10	99	97	95	96	96	97	95	94	93	92	96	92	93	96	93	84	95	93
11	99	99	95	97	97	98	97	95	95	93	98	95	95	97	94	89	96	94
12	99	100	97	97	98	98	97	96	96	93	98	95	96	97	95	92	96	95
13	99		98	99	98	99	98	96	96	95	98	97	96	98	96	97	98	97
14	100		98	99	98	99	98	97	96	96	99	97	97	99	96	97	98	98
15			99	99	99	99	99	98	97	96	100	98	97	100	97	97	99	98
16			100	100	100	100	99	98	98	98	100	98	98		98	98	99	98
17							99	99	99	99	100	99	99		99	98	100	99
18							99	99	99	99	100	100	99		99	99		100
19							99	100	100	99	100		100		100	99		
20							100		100	100	100					99		
21							100			100						100		
22																		
23																		
24																		
25																		

Note. P= Physical, C= Cognitive, E= Emotional
