# Supplementary material for: Measuring physical, cognitive, and emotional aspects of exhaustion with the BOSS II-short version – results from a representative population-based study in Germany
Source: BMC Public Health. 2022 Mar 24;22:579. doi: 10.1186/s12889-022-12961-z (PMC8943994; doi:10.1186/s12889-022-12961-z)
Supplement: Supplementary file 2 — Additional file 2: R Code. [file 12889_2022_12961_MOESM2_ESM.pdf]

Additional file 2

R Code

```
#install.packages("psych")
#install.packages("lavaan")
#install.packages("semTools")

library(psych)
library(lavaan)
library(semTools)

# read data
#https://ipip.ori.org/30FacetNEO-PI-RItems.htm
#https://osf.io/tbmh5/

full.data <- read.csv("data/IPIP300_N.csv")

#examine data
names(full.data)

# compute mean scores
full.data$NAng.full = rowMeans(full.data[c("NAng1","NAng2","NAng3","NAng4","NAng5","NAng6","NAng7","NAng8","NAng9","NAng10")], na.rm = T)
full.data$NAnx.full = rowMeans(full.data[c("NAnx1","NAnx2","NAnx3","NAnx4","NAnx5","NAnx6","NAnx7","NAnx8","NAnx9","NAnx10")], na.rm = T)
full.data$NDep.full = rowMeans(full.data[c("NDep1","NDep2","NDep3","NDep4","NDep5","NDep6","NDep7","NDep8","NDep9","NDep10")], na.rm = T)
full.data$NImm.full = rowMeans(full.data[c("NImm1","NImm2","NImm3","NImm4","NImm5","NImm6","NImm7","NImm8","NImm9","NImm10")], na.rm = T)
```

```
full.data$NSel.full =
rowMeans(full.data[c("NSel1","NSel2","NSel3","NSel4","NSel5","NSel6","NSel7","NSel8",
"NSel9","NSel10")], na.rm = T)

full.data$NVul.full =
rowMeans(full.data[c("NVul1","NVul2","NVul3","NVul4","NVul5","NVul6","NVul7","NV
ul8","NVul9","NVul10")], na.rm = T)

full.data$N.full    =
rowMeans(full.data[c("NAng.full","NAnx.full","NDep.full","NImm.full","NSel.full","NVul.f
ull")], na.rm = T)


# some basic descriptives

describe(full.data)

table(full.data$SEX)

table(full.data$AGE)


### EFA

fa.sort(fa(full.data[,1:60],6))


#CFA models full

#One factor model

N.one.model <- "

Neuro =~ NAng1 + NAng2 + NAng3 + NAng4 + NAng5 + NAng6 + NAng7 + NAng8 +
NAng9 + NAng10

      + NAnx1 + NAnx2 + NAnx3 + NAnx4 + NAnx5 + NAnx6 + NAnx7 + NAnx8 + NAnx9
+ NAnx10

      + NDep1 + NDep2 + NDep3 + NDep4 + NDep5 + NDep6 + NDep7 + NDep8 + NDep9
+ NDep10

      + NImm1 + NImm2 + NImm3 + NImm4 + NImm5 + NImm6 + NImm7 + NImm8 +
NImm9 + NImm10

      + NSel1 + NSel2 + NSel3 + NSel4 + NSel5 + NSel6 + NSel7 + NSel8 + NSel9 + NSel10

      + NVul1 + NVul2 + NVul3 + NVul4 + NVul5 + NVul6 + NVul7 + NVul8 + NVul9 +
NVul10

"


#correlated factor model
```

```
N.cor.model <- "

NAng =~ NAng1 + NAng2 + NAng3 + NAng4 + NAng5 + NAng6 + NAng7 + NAng8 +
NAng9 + NAng10

NAnx =~ NAnx1 + NAnx2 + NAnx3 + NAnx4 + NAnx5 + NAnx6 + NAnx7 + NAnx8 +
NAnx9 + NAnx10

NDep =~ NDep1 + NDep2 + NDep3 + NDep4 + NDep5 + NDep6 + NDep7 + NDep8 +
NDep9 + NDep10

NImm =~ NImm1 + NImm2 + NImm3 + NImm4 + NImm5 + NImm6 + NImm7 + NImm8 +
NImm9 + NImm10

NSel =~ NSel1 + NSel2 + NSel3 + NSel4 + NSel5 + NSel6 + NSel7 + NSel8 + NSel9 +
NSel10

NVul =~ NVul1 + NVul2 + NVul3 + NVul4 + NVul5 + NVul6 + NVul7 + NVul8 + NVul9 +
NVul10

"


#higher-order model

N.hio.model <- "

NAng =~ NAng1 + NAng2 + NAng3 + NAng4 + NAng5 + NAng6 + NAng7 + NAng8 +
NAng9 + NAng10

NAnx =~ NAnx1 + NAnx2 + NAnx3 + NAnx4 + NAnx5 + NAnx6 + NAnx7 + NAnx8 +
NAnx9 + NAnx10

NDep =~ NDep1 + NDep2 + NDep3 + NDep4 + NDep5 + NDep6 + NDep7 + NDep8 +
NDep9 + NDep10

NImm =~ NImm1 + NImm2 + NImm3 + NImm4 + NImm5 + NImm6 + NImm7 + NImm8 +
NImm9 + NImm10

NSel =~ NSel1 + NSel2 + NSel3 + NSel4 + NSel5 + NSel6 + NSel7 + NSel8 + NSel9 +
NSel10

NVul =~ NVul1 + NVul2 + NVul3 + NVul4 + NVul5 + NVul6 + NVul7 + NVul8 + NVul9 +
NVul10


Neuro =~ NAng + NAnx + NDep + NImm + NSel + NVul

"


#bi-factor model

N.bif.model <- "
```

```
NAng =~ NAng1 + NAng2 + NAng3 + NAng4 + NAng5 + NAng6 + NAng7 + NAng8 +
NAng9 + NAng10

NAnx =~ NAnx1 + NAnx2 + NAnx3 + NAnx4 + NAnx5 + NAnx6 + NAnx7 + NAnx8 +
NAnx9 + NAnx10

NDep =~ NDep1 + NDep2 + NDep3 + NDep4 + NDep5 + NDep6 + NDep7 + NDep8 +
NDep9 + NDep10

NImm =~ NImm1 + NImm2 + NImm3 + NImm4 + NImm5 + NImm6 + NImm7 + NImm8 +
NImm9 + NImm10

NSel =~ NSel1 + NSel2 + NSel3 + NSel4 + NSel5 + NSel6 + NSel7 + NSel8 + NSel9 +
NSel10

NVul =~ NVul1 + NVul2 + NVul3 + NVul4 + NVul5 + NVul6 + NVul7 + NVul8 + NVul9 +
NVul10


Neuro =~ NAng1 + NAng2 + NAng3 + NAng4 + NAng5 + NAng6 + NAng7 + NAng8 +
NAng9 + NAng10

    + NAnx1 + NAnx2 + NAnx3 + NAnx4 + NAnx5 + NAnx6 + NAnx7 + NAnx8 + NAnx9
+ NAnx10

    + NDep1 + NDep2 + NDep3 + NDep4 + NDep5 + NDep6 + NDep7 + NDep8 + NDep9
+ NDep10

    + NImm1 + NImm2 + NImm3 + NImm4 + NImm5 + NImm6 + NImm7 + NImm8 +
NImm9 + NImm10

    + NSel1 + NSel2 + NSel3 + NSel4 + NSel5 + NSel6 + NSel7 + NSel8 + NSel9 + NSel10

    + NVul1 + NVul2 + NVul3 + NVul4 + NVul5 + NVul6 + NVul7 + NVul8 + NVul9 +
NVul10


Neuro ~~ 0*NAng + 0*NAnx + 0*NDep + 0*NImm + 0*NSel + 0*NVul

"


#estimate models

fit.one  <- cfa(N.one.model , full.data , missing = "FIML")

fit.hio  <- cfa(N.hio.model , full.data , missing = "FIML")

fit.cor  <- cfa(N.cor.model , full.data , missing = "FIML")

fit.bif  <- cfa(N.bif.model , full.data , missing = "FIML", std.lv = T) #std.lv = T for
convergence reasons:  frees first loading and constrains factor variance to 1
```

```
#optional: plot models
#install.packages("semPlot")
#semPlot::semPaths(fit.cor)
#semPlot::semPaths(fit.hio)
#semPlot::semPaths(fit.bif)

#model fit comparison
round(rbind(
fitMeasures(fit.one, c("df","chisq","cfi","rmsea","srmr")),
fitMeasures(fit.hio, c("df","chisq","cfi","rmsea","srmr")),
fitMeasures(fit.cor, c("df","chisq","cfi","rmsea","srmr")),
fitMeasures(fit.bif, c("df","chisq","cfi","rmsea","srmr"))),3)

anova(fit.one,fit.hio,fit.cor,fit.bif)

#model parameters
summary(fit.one, standardized = T)
summary(fit.hio, standardized = T)
summary(fit.cor, standardized = T)
summary(fit.bif, standardized = T)

#reliability
round(reliability(fit.one) , 2)
round(reliability(fit.hio) , 2)
round(reliability(fit.cor) , 2)
round(reliability(fit.bif) , 2)

#measurement invariance example
fit.cor.config  <- cfa(N.cor.model , full.data , group = "SEX")
fit.cor.metric  <- cfa(N.cor.model , full.data , group = "SEX", group.equal = c("loadings"))
```

```
fit.cor.scalar  <- cfa(N.cor.model , full.data , group = "SEX", group.equal = c("loadings", "intercepts"))

fit.cor.strict  <- cfa(N.cor.model , full.data , group = "SEX", group.equal = c("loadings", "intercepts", "residuals"))


round(rbind(

fitMeasures(fit.cor.config,c("df","chisq","CFI","SRMR","RMSEA")),

fitMeasures(fit.cor.metric,c("df","chisq","CFI","SRMR","RMSEA")),

fitMeasures(fit.cor.scalar,c("df","chisq","CFI","SRMR","RMSEA")),

fitMeasures(fit.cor.strict,c("df","chisq","CFI","SRMR","RMSEA"))),3)


#number of positive / negative items

pos.items <- c("NAnx1", "NAnx2", "NAnx3", "NAnx4", "NAnx5", "NAng1", "NAng2", "NAng3", "NAng5", "NAng6", "NDep1", "NDep2", "NDep3", "NDep5", "NDep6", "NDep7", "NDep8", "NSel1", "NSel2", "NSel3", "NSel5", "NSel6", "NSel7", "NImm1", "NImm5", "NImm6", "NImm7", "NImm8", "NVul1", "NVul2", "NVul3", "NVul5", "NVul6")

neg.items <- c("NAnx6", "NAnx7", "NAnx8", "NAnx9", "NAnx10", "NAng4", "NAng7", "NAng8", "NAng9", "NAng10", "NDep4", "NDep9", "NDep10", "NSel4", "NSel8", "NSel9", "NSel10", "NImm2", "NImm3", "NImm4", "NImm9", "NImm10", "NVul4", "NVul7", "NVul8", "NVul9", "NVul10")


length(pos.items) #how many positively coded items in full form

length(neg.items) #how many negatively coded items in full form


# correlation with covariates (higher order model in this case because of multicolinearity)


N.ho.age.model <- "

NAng =~ NAng1 + NAng2 + NAng3 + NAng4 + NAng5 + NAng6 + NAng7 + NAng8 + NAng9 + NAng10

NAnx =~ NAnx1 + NAnx2 + NAnx3 + NAnx4 + NAnx5 + NAnx6 + NAnx7 + NAnx8 + NAnx9 + NAnx10

NDep =~ NDep1 + NDep2 + NDep3 + NDep4 + NDep5 + NDep6 + NDep7 + NDep8 + NDep9 + NDep10

NImm =~ NImm1 + NImm2 + NImm3 + NImm4 + NImm5 + NImm6 + NImm7 + NImm8 + NImm9 + NImm10
```

```
NSel =~ NSel1 + NSel2 + NSel3 + NSel4 + NSel5 + NSel6 + NSel7 + NSel8 + NSel9 + NSel10

NVul =~ NVul1 + NVul2 + NVul3 + NVul4 + NVul5 + NVul6 + NVul7 + NVul8 + NVul9 + NVul10


Neuro =~ NAng + NAnx + NDep + NImm + NSel + NVul


AGE ~ Neuro
"
fit.ho.age <- cfa(N.ho.age.model  , full.data )
summary(fit.ho.age, standardized = T)


############################################################################
#####
############### Creating an optimization function ##############################
############################################################################
#####


### Full model items
# full item list with items per factor
list.items <- list(
  NAng = c("NAng1","NAng2","NAng3","NAng4","NAng5","NAng6","NAng7","NAng8","NAng9","NAng10"),
  NAnx = c("NAnx1","NAnx2","NAnx3","NAnx4","NAnx5","NAnx6","NAnx7","NAnx8","NAnx9","NAnx10"),
  NDep = c("NDep1","NDep2","NDep3","NDep4","NDep5","NDep6","NDep7","NDep8","NDep9","NDep10"),
  NImm = c("NImm1","NImm2","NImm3","NImm4","NImm5","NImm6","NImm7","NImm8","NImm9","NImm10"),
  NSel = c("NSel1","NSel2","NSel3","NSel4","NSel5","NSel6","NSel7","NSel8","NSel9","NSel10"),
```

```
  NVul =
c("NVul1","NVul2","NVul3","NVul4","NVul5","NVul6","NVul7","NVul8","NVul9","NVul
10"))


#vector with all items (needed for pheromones)
item.vector <- unlist(list.items, use.names = F)


### Short model items
#3 example item selection of 4 items per factor (as would be selected by ACO)
selected.items.1 <- list(
  NAng = c("NAng1","NAng2","NAng3","NAng4"),
  NAnx = c("NAnx1","NAnx2","NAnx3","NAnx4"),
  NDep = c("NDep1","NDep2","NDep3","NDep4"),
  NImm = c("NImm1","NImm2","NImm3","NImm4"),
  NSel = c("NSel1","NSel2","NSel3","NSel4"),
  NVul = c("NVul1","NVul2","NVul3","NVul4"))


selected.items.2 <- list(
  NAng = c("NAng5","NAng6","NAng7","NAng8"),
  NAnx = c("NAnx5","NAnx6","NAnx7","NAnx8"),
  NDep = c("NDep5","NDep6","NDep7","NDep8"),
  NImm = c("NImm5","NImm6","NImm7","NImm8"),
  NSel = c("NSel5","NSel6","NSel7","NSel8"),
  NVul = c("NVul5","NVul6","NVul7","NVul8"))


selected.items.3 <- list(
  NAng = c("NAng3","NAng6","NAng9","NAng10"),
  NAnx = c("NAnx3","NAnx6","NAnx9","NAnx10"),
  NDep = c("NDep3","NDep6","NDep9","NDep10"),
  NImm = c("NImm3","NImm6","NImm9","NImm10"),
  NSel = c("NSel3","NSel6","NSel9","NSel10"),
  NVul = c("NVul3","NVul6","NVul9","NVul10"))
```

```
##### 1. Optimization function


#positive and negative items (used for optimization)

pos.items <- c("NAnx1", "NAnx2", "NAnx3", "NAnx4", "NAnx5", "NAng1", "NAng2", "NAng3", "NAng5", "NAng6", "NDep1", "NDep2", "NDep3", "NDep5", "NDep6", "NDep7", "NDep8", "NSel1", "NSel2", "NSel3", "NSel5", "NSel6", "NSel7", "NImm1", "NImm5", "NImm6", "NImm7", "NImm8", "NVul1", "NVul2", "NVul3", "NVul5", "NVul6")

neg.items <- c("NAnx6", "NAnx7", "NAnx8", "NAnx9", "NAnx10", "NAng4", "NAng7", "NAng8", "NAng9", "NAng10", "NDep4", "NDep9", "NDep10", "NSel4", "NSel8", "NSel9", "NSel10", "NImm2", "NImm3", "NImm4", "NImm9", "NImm10", "NVul4", "NVul7", "NVul8", "NVul9", "NVul10")


#names of factors (used to write model syntax)

factors <- c("NAnx", "NAng", "NDep","NSel", "NImm", "NVul") #factor names for lavaan model


#pick one item set

selected.items = selected.items.1

#if you want to go through the fit.function manually (i.e., execute each line)

data = full.data


### fit function start

fit.function = function(selected.items, data){


  tryCatch({ # used to ignore estimation errors, which would otherwise stop the entire ACO run


    ### write model syntax based on items

    model <- NULL


    # loop to specify each factor in the model

    for (fac in 1:length(factors)) {

      model <- paste(model, paste0(factors[fac], " =~ ", paste(selected.items[[fac]], collapse=" + ")), "\n") # write factor =~ items (separated by +); "\n" = new line
```

```
}

# possible model modifications:
#model <- paste0(model, paste0("G =~ ", paste(factors, collapse=" + "))) #higher-order model
#model <- paste0(model, paste0("G =~ ", paste(unlist(selected.items), collapse=" + "))) #bifactor model

# this is what the model looks like:
model

# estimate model
fit <- cfa(model = model, data = data, missing = "FIML")  #,orthogonal = TRUE, std.lv = TRUE) #required for bi-factor model (std.lv for convergence reasons)

### optimization #1: model fit
# note: I've added ( ) around most commands so you can see the output directly
(mod.fit <- fitMeasures(fit, c("cfi","rmsea","srmr")))

(phi.CFI   <- 1/(1+exp(50*(.90-mod.fit[1]))))       #logistic transformation of CFI with cutoff at .90 and "slope" of 50
(phi.RMSEA <- 1-(1/(1+exp(100*(.06-mod.fit[2])))))  #logistic transformation of RMSEA with cutoff at .06 and "slope" of 100
(phi.SRMR  <- 1-(1/(1+exp(100*(.06-mod.fit[3])))))  #logistic transformation of SRMR with cutoff at .06 and "slope" of 100

(phi.fit <- (phi.CFI + phi.RMSEA + phi.SRMR)/3) #mean value of transformed values

### optimization #2: reliability
(omega.all <- semTools::reliability(fit)[4,]) #extract hierarchical omega using reliability (semTools) function
(omega.mean <- mean(omega.all)) #compute average omega across all factors
```

```
(phi.rel <- 1/(1+exp(100*(.7-omega.mean)))) #logistic transformation of omega with cutoff at .70 and "slope" of 100

### optimization #3: correlation with full scale
(cor.full.all <- c(
cor(data$NAng.full , rowMeans(data[selected.items[[1]]], na.rm = T), use = "p"),
cor(data$NAnx.full , rowMeans(data[selected.items[[2]]], na.rm = T), use = "p"),
cor(data$NDep.full , rowMeans(data[selected.items[[3]]], na.rm = T), use = "p"),
cor(data$NImm.full , rowMeans(data[selected.items[[4]]], na.rm = T), use = "p"),
cor(data$NSel.full , rowMeans(data[selected.items[[5]]], na.rm = T), use = "p"),
cor(data$NVul.full , rowMeans(data[selected.items[[6]]], na.rm = T), use = "p")))

(cor.full.mean <- mean(cor.full.all))

(phi.cor <- 1/(1+exp(100*(.90-cor.full.mean)))) #logistic transformation of correlation with cutoff at .90 and "slope" of 100

### optimization #4: item key balance
(selected.vector <- unlist(selected.items, use.names = F)) # transform list into vector for comparison
(n.neg.items <- sum(is.element(selected.vector,neg.items))) #how items are negatively coded

(phi.bal <- 5*(1/sqrt(8*pi)*exp(-((n.neg.items-12)^2/8)))) # transformation into gaussian function with maximum (1) at 12 items

### end of function
# save detailed fit information;
(fit.detail <- round(c(mod.fit, omega.all, cor.full.all,n.neg.items),3))
#optional: give it nicer names for live feedback
names(fit.detail) <- c("cfi","rmsea","srmr",paste0(factors,".rel"),paste0(factors,".r"),"N neg")
```

```
    # compute overall optimization criterion ranging from 0 tp 1
    (phi.overall <- (phi.fit + phi.rel + phi.cor + phi.bal)/4)

    # return information to ACO
    return(list(as.numeric(phi.overall),
                fit.detail,
                model))

  }, # end of tryCatch
  warning = function(w) return(0), #optional, punishes negative residual variances etc.
  error = function(e) return(0)) #essential, deals with non-convergence

} #end fit function

######### 2. Evaluate some solutions

# compare the 3 item sets:
fit.function(selected.items.1, full.data)
fit.function(selected.items.2, full.data)
fit.function(selected.items.3, full.data)

# with random selection of items
selected.items.n <- list(
  NAng = sample(list.items[[1]], 4),
  NAnx = sample(list.items[[2]], 4),
  NDep = sample(list.items[[3]], 4),
  NImm = sample(list.items[[4]], 4),
  NSel = sample(list.items[[5]], 4),
  NVul = sample(list.items[[6]], 4))

fit.function(selected.items.n, full.data)
```

```
###### 3. Illustration of the logistic transformation
cfi <- seq(.7,1,.001)
rmsea <- seq(.15,0,-.001)
neg.item <- seq(0,24,1)

# Example CFI
#1/(1+exp(50*(.90-cfi)))) is the same as logistic(cfi, d=.90, a=50, c=0, z=1)
phi.cfi.90.50  <- logistic(cfi, d=.90, a=50, c=0, z=1) #logistic transformation of CFI with cutoff at .90 and "slope" of 50; transformed score from 0 to 1
phi.cfi.90.100 <- logistic(cfi, d=.90, a=100, c=0, z=1) #logistic transformation of CFI with cutoff at .90 and "slope" of 100; transformed score from 0 to 1
phi.cfi.95.50  <- logistic(cfi, d=.95, a=50, c=0, z=1) #logistic transformation of CFI with cutoff at .95 and "slope" of 100; transformed score from 0 to 1
phi.cfi.95.100 <- logistic(cfi, d=.95, a=100, c=0, z=1) #logistic transformation of CFI with cutoff at .95 and "slope" of 100; transformed score from 0 to 1

 plot(cfi,phi.cfi.90.50, type = "l", ylim = c(0,1))
lines(cfi,phi.cfi.90.100, lty = 2)
lines(cfi,phi.cfi.95.50, col = "red")
lines(cfi,phi.cfi.95.100, col = "red", lty = 2)

### Example RMSEA (1 - so that lower values are better)
phi.rmsea.06.50  <- 1 - logistic(rmsea, d=.06, a=50, c=0, z=1)
phi.rmsea.06.100 <- 1 - logistic(rmsea, d=.06, a=100, c=0, z=1)
phi.rmsea.08.50  <- 1 - logistic(rmsea, d=.08, a=50, c=0, z=1)
phi.rmsea.08.100 <- 1 - logistic(rmsea, d=.08, a=100, c=0, z=1)

 plot(rmsea,phi.rmsea.06.50 , type = "l", ylim = c(0,1))
lines(rmsea,phi.rmsea.06.100, lty = 2)
lines(rmsea,phi.rmsea.08.50 , col = "red")
lines(rmsea,phi.rmsea.08.100, col = "red", lty = 2)
```

```
### Example of item key balance transformation into gaussian function
phi.bal <- 5*(1/sqrt(8*pi)*exp(-((neg.item-12)^2/10)))

plot(neg.item, phi.bal , type = "l", ylim = c(0,1))

#alternatively you can "mirror" the logistic transformation by optimizing the absolute
difference to a target number of items:
phi.bal.log  <- 1 - logistic(abs(neg.item-12), d=2, a=2, c=0, z=1)
lines(neg.item, phi.bal.log , col = "green")

phi.bal.log  <- 1 - logistic(abs(neg.item-12), d=4, a=1, c=0, z=1)
lines(neg.item, phi.bal.log , col = "red")

 library(psych)
library(lavaan)
library(semTools)

###########################################################################
################################# Basic ACO #############################
###########################################################################

### 1. create fit function (in this case CFI and RMSEA)

fit.function = function(selected.items, data){

  tryCatch({ # used to ignore estimation errors, which would otherwise stop the entire ACO
run

  model <- NULL #create lavan model
  for(fac in 1:length(factors)){ #for each factor write factor =~ items
    model <- paste(model, factors[fac], "=~ ", paste(selected.items[[fac]], collapse = " + "),
"\n")
```

```
  }

  print(model) # print model while running

  fit <- cfa(model, data, missing = "FIML") #estimate model

  CFI   <- fitMeasures(fit, "cfi") #extract CFI
  RMSEA <- fitMeasures(fit, "rmsea") #extract RMSEA

  phi.CFI <- 1/(1+exp(100*(.90-CFI)))  #logistic transformation of CFI with turning point at .90 and "slope" of 100
  phi.RMSEA <- 1-(1/(1+exp(100*(.06-RMSEA)))) #logistic transformation of RMSEA with turning point at .06 and "slope" of 100

  phi.overall <- (phi.CFI + phi.RMSEA)/2 #mean value of both transformed values
  fit.detail <- c(CFI,RMSEA) #also save detailed information

  }, # end of tryCatch
  #warning = function(w) return(0), #optional, punishes negative residual variances etc.
  error = function(e) return(0)) #essential, deals with non-convergence

  return(list(phi.overall,
              fit.detail,
              model))
}

### 2. required arguments for ACO: names of factors, items, number of items desired, number of ants and iterations, evaporation rate, names for output files

#item names organized by factor
list.items <- list(
```

```
  NAng = 
c("NAng1","NAng2","NAng3","NAng4","NAng5","NAng6","NAng7","NAng8","NAng9","
NAng10"),

  NAnx = 
c("NAnx1","NAnx2","NAnx3","NAnx4","NAnx5","NAnx6","NAnx7","NAnx8","NAnx9","
NAnx10"),

  NDep = 
c("NDep1","NDep2","NDep3","NDep4","NDep5","NDep6","NDep7","NDep8","NDep9","N
Dep10"),

  NImm = 
c("NImm1","NImm2","NImm3","NImm4","NImm5","NImm6","NImm7","NImm8","NImm9
","NImm10"),

  NSel = 
c("NSel1","NSel2","NSel3","NSel4","NSel5","NSel6","NSel7","NSel8","NSel9","NSel10"),

  NVul = 
c("NVul1","NVul2","NVul3","NVul4","NVul5","NVul6","NVul7","NVul8","NVul9","NVul
10"))


#factor names (for lavaan)

factors <- c("NAng","NAnx","NDep","NImm","NSel","NVul")


#desired number of items per factor

i.per.f <- c(4,4,4,4,4,4)


#desired number of ants per iterations and iterations (after a new best solution has been found)

ants <- 10

max.iter <- 10


#evaporation of pheromones after each iteration (multiplied with pheromones each iteration)

evaporation <- .95 #Empfehlung: .95 bis .99


#summaryfiles to save the search results

summaryfile.all <- "ACO_S4_4_all.csv" #for all models

summaryfile.fin <- "ACO_S4_4_best.csv" #for best model
```

```
#add titles to summary files
item.vector <- unlist(list.items, use.names = F)

#for all models (optional, but useful to see pheromones)
title.all <- matrix(c(item.vector,"Run", "Iteration", "Ant", "Phi.overall", "CFI" , "RMSEA" , paste0(item.vector,".PH")), 1)
write.table(title.all, file = summaryfile.all, append = T,quote = F, sep = ";", row.names = F, col.names = F)

#for best model
title.fin <- matrix(c(item.vector, "Phi.overall", "CFI", "RMSEA"), 1) #change title if you add more criteria (names need to match fit.detail)
write.table(title.fin, file = summaryfile.fin, append = T,quote = F, sep = ";", row.names = F, col.names = F)

### 3. ACO funtion

#needed if you want to go through the function manually (i.e., execute one line at a time)
#data = full.data
#ant = 1
#fac = 1

#ACO function start
ACO <- function(data, list.items, factors, i.per.f, ants, max.iter, evaporation, summaryfile.all, summaryfile.fin) {

  best.phi.overall <- 0 #starting value for best fit within iteration
  best.so.far.phi.overall <- 0 #starting value for best fit across all iterations

  #note: I pus some commands in ( ) so you can see the output/values directly
  (item.vector <- unlist(list.items, use.names = F)) #used for output and number of items in total
  (full <- length(item.vector)) #number of items in total (for pheromones)
```

```
#creates the initial pheromone levels = 1
(pheromones <- rep(1, full))

#creates a list to store items per factor
selected.items <- list.items

#starts counting the iterations (resetable iteration counter)
iter <- 1

#starts counting continuous runs regardless of result.
run <- 1

#starts loop through iterations.
while (iter <= max.iter) { #do not run this line if you go through the function manually

  #sends a number of ants per time.
  for(ant in 1:ants){ #do not run this line if you go through the function manually

    #selects items for all factors.
    for (fac in 1:length(factors)) { #run this loop to select items though

      #selects the items for a short form for the factor
      (positions <- is.element(item.vector, list.items[[fac]])) #identify pheromone levels of
items
      (prob <- pheromones[positions] / sum(pheromones[positions])) #determine probability
of items to be selected based on pheromones
      (items <- sample(list.items[[fac]], size = i.per.f[fac], prob = prob))

      #stores selected items for factor in list
      selected.items[[fac]] <- items
```

```
} #finishes factor loop

#creates a vector of selected items.
(selected.vector <- unlist(selected.items, use.names = F))

#creates a 0/1 vector of the same length of the full form indicating
#whether an item was selected or not for the short form.
(solution <- is.element(item.vector,selected.vector))

#evaluate item selection
(item.eval <- fit.function(selected.items,data))

#extract overall fit
phi.overall <- item.eval[[1]]

#in case of non-convergence, this part is skipped
if(phi.overall != 0){

  #extract detailed fit statistics
  fit.detail <- item.eval[[2]]

  #print fit statistics
  print(round(fit.detail,3))

  #save information in summary file
  (fit.save <- matrix(c(solution, run, iter, ant, phi.overall, fit.detail, round(pheromones,2)), 1))
  write.table(fit.save, file = summaryfile.all, append = T, quote = F, sep = ";", row.names = F, col.names = F)
}

#if the new solution is the best solution WITHIN the iteration, update best solution
```

```
    if (phi.overall > best.phi.overall) {

      # updates solution.
      best.phi.overall <- phi.overall #best evaluation score
      best.solution <- solution #which items have been selected as 0/1 vector
      best.items <- selected.items #items as list
      best.fit.detail <- fit.detail #deteiled psychometrics

    }

  }#ends loop through ants (= one iteration)

  # reduce pheromones by evaporation value
  pheromones <- pheromones*evaporation

  #updates pheromone for best solution after each iteration by overall evaluation score (ranging from 0 to 1)
  (pheromones <- pheromones + best.phi.overall*best.solution)

  #if the new solution is the best solution ACROSS ALL iterations, update best overall solution
  if (best.phi.overall > best.so.far.phi.overall) {

    #updates best so far solution and fit information
    best.so.far.solution <- best.solution
    best.so.far.items <- best.items
    best.so.far.phi.overall <- best.phi.overall
    best.so.far.fit.detail <- best.fit.detail

    #re-starts count.
    iter <- 1
```

```
      #end if clause for pheromone adjustment.
    } else {

      #advances count.
      iter <- iter + 1

    }

    #ends loop.
    run <- run + 1
  } #end of all iterations

  # Compile a matrix with the final solution.
  final.solution <- matrix(c(best.so.far.solution, best.so.far.phi.overall, best.so.far.fit.detail),1)
  write.table(final.solution, file = summaryfile.fin, append = T,quote = F, sep = ";", row.names
= F, col.names = F)

  print(paste0("The best solution achieved a score of: ", round(best.so.far.phi.overall,2)))
  print(round(best.so.far.fit.detail,3))

  #return best model (for items and optimization values see summaryfile)
  return(best.so.far.items)
}

####################### 4. Run ACO

set.seed(1) #ensures that run replicates
short.model <- ACO(full.data, list.items, factors, i.per.f, ants, max.iter, evaporation,
summaryfile.all, summaryfile.fin)

short.model
```

```
# you can use the item list in short.model to run the fit function again:
(final.solution <- fit.function(selected.items = short.model, data = full.data))

# and use the model provided by the fit.function to examine the final solution in more detail
final.model <- final.solution[[3]]
fit.final <- cfa(final.model, full.data, missing = "FIML")
summary(fit.final, standardized = T, fit.measures = T)

library(foreign)
library(stuart)
library(semTools)

df <- read.spss(file="R19_SCHMALB_nomiss_nounderage.SAV", to.data.frame = T,
max.value.labels = 0)

#Randomly split df
n <- 2429
data <- data.frame(x=runif(n), y=rnorm(n))
ind <- sample(c(TRUE, FALSE), n, replace=TRUE, prob=c(0.5, 0.5))
df_efa <- df[ind, ]
df_cfa <- df[!ind, ]

cor(df[,132:134], df[,132:134])

#stuart scale shortening
fs <- list(BODY = c("qh101", "qh102", "qh103", "qh104", "qh105", "qh106", "qh107",
"qh108", "qh109", "qh110"),
        COG = c("qh111", "qh112", "qh113", "qh114", "qh115", "qh116", "qh117", "qh118",
"qh119", "qh120"),
        EMO = c("qh121", "qh122", "qh123", "qh124", "qh125", "qh126", "qh127", "qh128",
"qh129", "qh130"))
```

```
ni <- list(5, 5, 5)

combinations(df_efa, fs, ni)

sel <- mmas(df_efa, fs, ni, grouping = "s2", group.invariance = "strong")

summary(sel)

summary(sel$final, fit.measures=T)


#CFA


BOSS_Model <- "BODY =~ qh101 + qh102 + qh103 + qh104 + qh105 + qh106 + qh107 + qh108 + qh109 + qh110
	COG =~ qh111 + qh112 + qh113 + qh114 + qh115 + qh116 + qh117 + qh118 + qh119 + qh120
	EMO =~ qh121 + qh122 + qh123 + qh124 + qh125 + qh126 + qh127 + qh128 + qh129 + qh130"


BOSS_Model <- "BODY =~ qh105 + qh106 + qh108 + qh109 + qh110
	COG =~ qh113 + qh115 + qh116 + qh118 + qh119
	EMO =~ qh121 + qh123 + qh124 + qh125 + qh126"



fit <- cfa(BOSS_Model, data=df_cfa, estimator="MLM")


summary(fit, fit.measures=T)

inspect(fit, what="std")

reliability(fit)


#measurement invariance-----

a < -measurementInvariance(model=BOSS_Model, data=df, estimator="MLM", strict=T, group="age_group", fit.measures = c("chisq.scaled","cfi.robust"))

b <- partialInvariance(a, type="strict")


fit <- cfa(BOSS_Model, data=df[df_cfa$age_group==7,], estimator="MLM")

summary(fit, fit.measures=T)
```

```
moreFitIndices(fit)[4]


fit <- cfa(BOSS_Model, data=df, estimator="MLM", group = "age_group")

fit <- cfa(BOSS_Model, data=df, estimator="MLM", group = "age_group",
group.equal="loadings")

fit <- cfa(BOSS_Model, data=df, estimator="MLM", group = "age_group",
group.equal=c("loadings","intercepts"))

fit <- cfa(BOSS_Model, data=df, estimator="MLM", group = "age_group",
group.equal=c("loadings","intercepts"),

        group.partial = c("qh106~1", "qh110~1"))

fit <- cfa(BOSS_Model, data=df, estimator="MLM", group = "age_group",
group.equal=c("loadings","intercepts","residuals"),

        group.partial = c("qh106~1", "qh110~1", "qh105~~qh105", "qh106~~qh106",
"qh110~~qh110", "qh113~~qh113",

                    "qh116~~qh116", "qh118~~qh118", "qh119~~qh119"))


#correlations----

cor(df[c("BOSS_Physical", "BOSS_Cognitive", "BOSS_Emotional", "BOSS30_Physical",
"BOSS30_Cognitive", "BOSS30_Emotional",

     "euro_qol", "phq_9", "gad_7")])


#sociodemographic table

table(df$s2)

table(df$age_group)

table(df$education)

table(df$family)

table(df$work)

table(df$income)

mean(df$Alter)

sd(df$Alter)


aggregate(BOSS_Physical ~ s2, df, function(x) c(mean = mean(x), sd = sd(x)))

aggregate(BOSS_Cognitive ~ s2, df, function(x) c(mean = mean(x), sd = sd(x)))
```

```
aggregate(BOSS_Emotional ~ s2, df, function(x) c(mean = mean(x), sd = sd(x)))

aggregate(BOSS_Physical ~ age_group, df, function(x) c(mean = mean(x), sd = sd(x)))
aggregate(BOSS_Cognitive ~ age_group, df, function(x) c(mean = mean(x), sd = sd(x)))
aggregate(BOSS_Emotional ~ age_group, df, function(x) c(mean = mean(x), sd = sd(x)))

aggregate(BOSS_Physical ~ education, df, function(x) c(mean = mean(x), sd = sd(x)))
aggregate(BOSS_Cognitive ~ education, df, function(x) c(mean = mean(x), sd = sd(x)))
aggregate(BOSS_Emotional ~ education, df, function(x) c(mean = mean(x), sd = sd(x)))

aggregate(BOSS_Physical ~ family, df, function(x) c(mean = mean(x), sd = sd(x)))
aggregate(BOSS_Cognitive ~ family, df, function(x) c(mean = mean(x), sd = sd(x)))
aggregate(BOSS_Emotional ~ family, df, function(x) c(mean = mean(x), sd = sd(x)))

aggregate(BOSS_Physical ~ work, df, function(x) c(mean = mean(x), sd = sd(x)))
aggregate(BOSS_Cognitive ~ work, df, function(x) c(mean = mean(x), sd = sd(x)))
aggregate(BOSS_Emotional ~ work, df, function(x) c(mean = mean(x), sd = sd(x)))

aggregate(BOSS_Physical ~ income, df, function(x) c(mean = mean(x), sd = sd(x)))
aggregate(BOSS_Cognitive ~ income, df, function(x) c(mean = mean(x), sd = sd(x)))
aggregate(BOSS_Emotional ~ income, df, function(x) c(mean = mean(x), sd = sd(x)))

#norm values----
#go through s2[sex] and age groups

cumsum(table(df$BOSS_Physical[df$s2==1 & df$age_group==2]))/sum(table(df$BOSS_Physical[df$s2==1 & df$age_group==2]))
cumsum(table(df$BOSS_Cognitive[df$s2==1 & df$age_group==2]))/sum(table(df$BOSS_Cognitive[df$s2==1 & df$age_group==2]))
cumsum(table(df$BOSS_Emotional[df$s2==1 & df$age_group==2]))/sum(table(df$BOSS_Emotional[df$s2==1 & df$age_group==2]))
```
